# Registration under the New Medical Law

**Published:** 1880-12

**Authors:** 


### THE REGISTRATION UNDER THE NEW MEDICAL LAW.

THE following additions only have been made to the list of physicians who have registered in the Erie County Clerk's office since the issue of the November number of the JOURNAL.

| NAME. | GRADUATE OF | DATE. |
|---|---|---|
| Atwood, Harley L. | Buffalo University | Feb., 1872. |
| Clark, Edward | University of Buffalo | Feb. 25, 1 80. |
| Caulkings, Frank | Student in Homeopathic College of Physicians and Surgeons at Buffalo, and intending to graduate within lawful time, signed S. W. Wetmore | 1880. |
| Fullerton, Mrs. M. A. | Philadelphia Univ. of Medicine and Surgery | June, 1878. |
| Henne, Christoph | Buffalo Homeopathic Medical Society | May, 1876. |
| Hinkley, Alonzo S. | Western College of Homeopathic Medicine, Cleveland, O. | March, 1856. |
| Lapham, Geo. H. | Jefferson Medical College, Pa | March, 1836. |
| Outwater, Samuel | University of the City of New York | Mar. 12, 1879. |
| Sovereign, Baxter | Hospital College, Cleveland, O. | 1869. |
| Trull, Hiram P. | Buffalo University | Feb. 22, 1868. |
| Wright, A. R. | Cleveland Homeopathic Hospital College | Mar. 10, 1857. |

